# Supplementary material for: Infective Endocarditis in Belgium: Prospective Data in Adults from the ESC EORP European Endocarditis Registry
Source: J Clin Med. 2024 Feb 28;13(5):1371. doi: 10.3390/jcm13051371 (PMC10932099; doi:10.3390/jcm13051371)
Supplement: Supplementary file 1 [file jcm-13-01371-s001.zip › jcm-2850595-supplementary.pdf]

Supplementary material

Supplementary Table S1. Definitions of the terms used in the European Society of Cardiology 2015 modified criteria for the diagnosis of infective endocarditis.

Major criteria<br><br>1. Blood cultures positive for IE<br>a. Typical microorganisms consistent with IE from 2 separate blood cultures:<br>– Viridans streptococci, *Streptococcus gallolyticus (S bovis),* HACEK group, *S. aureus*; or<br>– Community-acquired enterococci, in the absence of a primary focus; or<br>b. Microorganisms consistent with IE from persistently positive blood cultures:<br>– $\geq$2 positive blood cultures of blood samples drawn >12 h apart; or<br>– All of 3 or a majority of $\geq$4 separate cultures of blood (with first and last samples drawn $\geq$1 h apart); or<br>c. Single positive blood culture for *Coxiella burnetii* or phase I IgG antibody titre >1:800<br><br>2. Imaging positive for IE<br>a. Echocardiogram positive for IE:<br>– Vegetation;<br>– Abscess, pseudoaneurysm, intracardiac fistula;<br>– Valvular perforation or aneurysm<br>– New partial dehiscence of prosthetic valve<br>b. Abnormal activity around the site of prosthetic valve implantation detected by 18F-FDG PET/CT (only if the prosthesis was implanted for >3 months) or radiolabelled leukocytes SPECT/CT<br>c. Definite paravalvular lesions by cardiac CT
Minor criteria<br>1. Predisposition such as predisposing heart condition, or injection drug use<br>2. Fever defined as temperature >38°C<br>3. Vascular phenomena (including those detected only by imaging): major arterial emboli, septic pulmonary infarcts, infectious (mycotic) aneurysm, intracranial haemorrhage, conjunctival haemorrhages, and Janeway's lesions<br>4. Immunological phenomena: glomerulonephritis, Osler's nodes, Roth's spots, and rheumatoid factor

5. Microbiological evidence: positive blood culture but does not meet a major criterion as noted above or serological evidence of active infection with organism consistent with IE

Supplementary Table S2. Clinical presentation of Belgian infective endocarditis patients.

	Total (n = 127)	NVE (n = 75)	PRVE (n = 45)	CDRIE (n = 7)	*P*-value
Signs and symptoms					
Fever	89/127 (70.1%)	49/75 (65.3%)	35/45 (77.8%)	5/7 (71.4%)	0.390
Cardiac murmur	63/127 (49.6%)	43/75 (57.3%)	19/45 (42.2%)	1/7 (14.3%)	0.047*
Dizziness	26/127 (20.5%)	19/75 (25.3%)	5/45 (11.1%)	2/7 (28.6%)	0.113
Congestive heart failure	23/127 (18.1%)	12/75 (16.0%)	8/45 (17.8%)	3/7 (42.9%)	0.201
Shortness of breath	23/127 (18.1%)	12/75 (16.0%)	9/45 (20.0%)	2/7 (28.6%)	0.515
Cough	12/127 (9.4%)	6/75 (8.0%)	4/45 (8.9%)	2/7 (28.6%)	0.201
Septic shock	12/127 (9.4%)	7/75 (9.3%)	4/45 (8.9%)	1/7 (14.3%)	0.775
Chest pain	7/127 (5.5%)	6/75 (8.0%)	1/45 (2.2%)	0/7 (0.0%)	0.500
Syncope	5/127 (3.9%)	3/75 (4.0%)	2/45 (4.4%)	0/7 (0.0%)	1.000
Janeway lesions	5/127 (3.9%)	2/75 (2.7%)	3/45 (6.7%)	0/7 (0.0%)	0.519
Osler's nodes	4/127 (3.1%)	3/75 (4.0%)	1/45 (2.2%)	0/7 (0.0%)	1.000
Cerebrovascular accident	3/127 (2.4%)	2/75 (2.7%)	1/45 (2.2%)	0/7 (0.0%)	1.000
Cardiogenic shock	2/127 (1.6%)	1/75 (1.3%)	1/45 (2.2%)	0/7 (0.0%)	1.000
Roth spots	2/127 (1.6%)	2/75 (2.7%)	0/45 (0.0%)	0/7 (0.0%)	0.580
Complications at admission					
Embolic events	33/127 (26%)	25/75 (33.3%)	8/45 (17.8%)	0/7 (0.0%)	0.046*
Pulmonary	3/127 (2.4%)	3/75 (4.0%)	0/45 (0.0%)	0/7 (0.0%)	0.404
Cerebral	19/127 (15.0%)	15/75 (20.0%)	4/45 (8.9%)	0/7 (0.0%)	0.169
Splenic	3/127 (2.4%)	2/75 (2.7%)	1/45 (2.2%)	0/7 (0.0%)	1.000
Coronary	1/127 (0.8%)	1/75 (1.3%)	0/45 (0.0%)	0/7 (0.0%)	1.000
Renal	4/127 (3.1%	4/75 (5.3%)	0/45 (0.0%)	0/7 (0.0%)	0.437
Hepatic	0/127 (0.0%)	0/75 (0.0%)	0/45 (0.0%)	0/7 (0.0%)	1.000
Peripheral	3/127 (2.4%)	2/75 (2.7%)	1/45 (2.2%)	0/7 (0.0%)	1.000

	Total<br>(n = 127)	NVE<br>(n = 75)	PRVE<br>(n = 45)	CDRIE<br>(n = 7)	*P*-value
Abscess	17/127 (13.4%)	13/75 (17.3%)	3/45 (6.7%)	1/6 (14.3%)	0.201
Spondylitis	11/127 (8.7%)	7/75 (9.3%)	4/45 (8.9%)	0/7 (0.0%)	1.000
Haemorrhagic Stroke	0/127 (0.0%)	0/75 (0.0%)	0/45 (0.0%)	0/7 (0.0%)	1.000

CDRIE, cardiac device related infective endocarditis; NVE, native valve infective endocarditis; PRVE, prosthetic or repaired valve infective endocarditis. A value of $P < 0.050$ was considered significant. (*) denotes significant *P*-values.

Supplementary Table S3. Microbiology in Belgian infective endocarditis patients.

	Total (n = 110)	NVE (n = 64)	PRVE (n = 39)	CDRIE (n = 7)	*P*-value
Methi-S S. aureus	37/110 (33.6%)	21/64 (32.8%)	12/39 (32.4%)	4/7 (57.1%)	0.440
Methi-R S. aureus	4/110 (3.6%)	3/64 (4.7%)	0/39 (0%)	1/7 (14.3%)	0.091
Methi-S Staph. coagulase negative	7/110 (6.4%)	3/64 (4.7%)	4/39 (10.3%)	0/7 (0.0%)	0.638
Methi-R Staph. coagulase negative	6/110 (5.5%)	1/64 (1.6%)	5/39 (12.8%)	0/7 (0.0%)	0.083
S. viridans	17/110 (15.5%)	13/64 (20.3%)	3/39 (7.7%)	1/7 (14.3%)	0.197
Enterococcus	17/110 (15.5%)	6/64 (9.4%)	9/39 (23.1%)	2/7 (28.6%)	0.072
S. gallolyticus	9/110 (8.2%)	6/64 (9.4%)	3/39 (7.7%)	0/7 (0.0%)	1.000
Gram negative bacillus	3/110 (2.7%)	1/64 (1.6%)	2/39 (5.1%)	0/7 (0.0%)	0.637
C. burnetii IgG anti phase I	0/127 (0.0%)	0/75 (0.0%)	0/45 (0.0%)	0/7 (0.0%)	-

Methi-R, methicilline-resistant; Methi-S, methicilline-sensitive. A value of $P < 0.050$ was considered significant.

Supplementary Table S4. In-hospital complications under therapy in Belgian infective endocarditis patients.

	Total (n =127)	NVE (n = 75)	PRVE (n = 45)	CDRIE (n = 7)	*P*-value
**Complications under therapy**					
Acute renal failure	46/127 (36.2%)	24/75 (32.0%)	19/45 (42.2%)	3/7 (42.9%)	0.459
Embolic events	30/127 (23.6%)	21/75 (28.0%)	9/45 (20.0%)	0/7 (0.0%)	0.211
Pulmonary	4/30 (13.3%)	2/21 (9.5%)	2/9 (22.2%)	-	0.563
Cerebral	14/30 (46.7%)	10/21 (47.6%)	4/9 (44.4%)	-	1.000
TIA	2/25 (8.0%)	2/18 (11.1%)	0/7 (0.0%)	-	1.000
Stroke	8/25 (32.0%)	5/18 (27.8%)	3/7 (42.9%)	-	0.640
Spleen	5/30 (16.7%)	3/21 (14.3%)	2/9 (22.2%)	-	0.622
Coronary	2/30 (6.7%)	1/21 (4.8%)	1/9 (11.1%)	-	0.517
Renal	6/30 (20.0%)	5/21 (23.8%)	1/9 (11.1%)	-	0.637
Hepatic	1/30 (3.3%)	1/21 (4.8%)	0.9 (0.0%)	-	1.000
Peripheral	1/30 (3.3%)	1/21 (4.8%)	0/9 (0.0%)	-	1.000
Positive blood cultures after 48h	26/127 (20.5%)	13/75 (17.3%)	11/45 (24.4%)	2/7 (28.6%)	0.511
Thrombopenia (<100000)	20/127 (15.7%)	13/75 (17.3%)	6/45 (13.3%)	1/7 (14.3%)	0.849
Spondylitis	15/127 (11.8%)	9/75 (12.0%)	6/45 (13.3%)	0/7 (0.0%)	0.909
Septic shock	15/127 (11.8%)	7/75 (9.3%)	6/45 (13.3%)	2/7 (28.6%)	0.216
Increasing vegetation size	12/127 (9.4%)	6/75 (8.0%)	6/45 (13.3%)	0/7 (0.0%)	0.437
CHF	11/127 (8.7%)	8/75 (10.7%)	3/45 (6.7%)	0/7 (0.0%)	0.754
Glomerulonephritis	11/127 (8.7%)	9/75 (12.0%)	2/45 (4.4%)	0/7 (0.0%)	0.338
Persistent fever (>7 days)	9/127 (7.1%)	4/75 (5.3%)	4/45 (8.9%)	1/7 (14.3%)	0.315
Cardiogenic shock	7/127 (5.5%)	4/75 (5.3%)	3/45 (6.7%)	0/7 (5.5%)	1.000
New abscess	7/127 (5.5%)	4/75 (5.3%)	3/45 (6.7%)	0/7 (0.0%)	1.000
AV block	5/127 (3.9%)	3/75 (4.0%)	2/45 (4.4%)	0/7 (0.0%)	1.000
Mycotic aneurysm	4/127 (3.1%)	2/75 (2.7%)	2/45 (4.4%)	0/7 (0.0%)	0.698
Cerebral Haemorrhage	1/127 (0.8%)	0/75 (0.0%)	1/45 (2.2%)	0/7 (0.0%)	0.413

	Total (n =127)	NVE (n = 75)	PRVE (n = 45)	CDRIE (n = 7)	*P*-value
Haemorrhagic Stroke	0/127 (0.0%)	0/75 (0.0%)	0/45 (0.0%)	0/7 (0.0%)	1.000

CDRIE, cardiac device related infective endocarditis; CHF, Congestive Heart Failure; NVE, native valve infective endocarditis; PRVE, prosthetic or repaired valve infective endocarditis. A value of $P < 0.050$ was considered significant.

Supplementary Table S5. Univariate Cox regression analysis for all causes of death in hospital in Belgian IE patients.

	Hazard Ratio	95% CI	*P*-value
Type of endocarditis: prosthesis + repair	2.27	[0.87-5.96]	0.096
Source of infection: community acquired	1.18	[0.14-9.59]	0.880
Age < 65 yo	0.62	[0.24-1.63]	0.806
Age 65 - 79 yo	1.32	[0.53-3.25]	0.335
Age >= 80 yo	1.85	[0.40-8.56]	0.553
Gender: male	0.98	[0.37-2.59]	0.964
Charlson index	1.18	[1.04-1.34]	0.011*
Creatinine >2mg/dl	3.49	[1.34-9.08]	0.011*
S. aureus	2.38	[0.88-6.49]	0.089
Congestive heart failure	0.84	[0.28-2.56]	0.757
Vegetation size > 10 mm	2.31	[0.28-19.28]	0.439
Cerebral complication	1.71	[0.28-10.55]	0.564
Abscess	1.13	[0.32-4.00]	0.853
Indication for surgery - not performed	13.72	[1.56-120.61]	0.018*
Indication for surgery - performed	3.34	[0.43-25.93]	0.018*

yo: years old. A value of $P < 0.050$ was considered significant. (*) denotes significant *P*-values.

Complete list of the EURO-ENDO Investigators Group and EURO-ENDO National Coordinators

EORP Oversight Committee: C.P. Gale, GB (Chair); B. Beleslin, RS; A. Budaj, PL; O. Chioncel, RO; N. Dagres, DE; N. Danchin, FR; J. Emberson, GB; D. Erlinge, SE; M. Glikson, IL; A. Gray, GB; M. Kayikcioglu, TR; A.P. Maggioni, IT; V.K. Nagy, HU; A. Nedoshivin, RU; A-S. Petronio, IT; J. Roos-Hesselink, NL; L. Wallentin, SE; U. Zeymer, DE.

Executive Committee: G. Habib, FR (Chair); P. Lancellotti, BE (Chair); B. Cosyns, BE; E. Donal, FR; P. Erba, IT; B. Iung, FR; A.P. Maggioni, IT; B.A. Popescu, RO; B. Prendergast, GB; P. Tornos, ES.

EORP Team: M. Andarala, C. Berle, A. Brunel-Lebecq, E. Fiorucci, C. Laroche, V. Missiamenou, C. Taylor.

National Coordinators: N.N. Ali Tatar-Chentir, DZ; M. Al-Mallah, SA; M. Astrom Aneq, SE; G. Athanassopoulos, GR; L.P. Badano, IT; S. Benyoussef, TN; E. Calderon Aranda, MX; N.M. Cardim, PT; K-L. Chan, CA; B. Cosyns, BE; I. Cruz, PT; T. Edvardsen, NO; G. Goliasch, AT; G. Habib, FR; A. Hagendorff, DE; K. Hristova, BG; B. Iung, FR; O. Kamp, NL; D-H. Kang, KR; W. Kong, SG; S. Matskeplishvili, RU; M. Meshaal, EG; M. Mirocevic, ME; A.N. Neskovic, RS; M. Pazdernik, CZ; E. Plonska-Gosciniak, PL; B.A. Popescu, RO; B. Prendergast, GB; M. Raissouni, MA; R. Ronderos, AR; L.E. Sade, TR; A. Sadeghpour, IR; A. Sambola, ES; S. Sengupta, IN; J. Separovic-Hanzevacki, HR; M. Takeuchi, JP; E. Tucay, PH; A.C. Tude Rodrigues, BR; A. Varga, HU; J. Vaskelyte, LT; K. Yamagata, MT; K. Yiangou, CY; H. Zaky, AE.

Investigators:

Argentina:Buenos Aires: I. Granada, M. Mahia, S. Ressi, F. Nacinovich, A. Iribarren, P. Fernandez Oses, G. Avegliano, E. Filipini, Corrientes: R. Obregon, M. Bangher, J. Dho, La Plata: L. Cartasegna, M.L. Plastino, V. Novas, C. Shigel, Florencio Varela: G. Reyes, M. De Santos, N. Gastaldello, M. Granillo Fernandez, M. Potito, G. Streitenberger, P. Velazco, J.H. Casabé, C. Cortes, E. Guevara, F. Salmo, M. Seijo, Austria:Vienna: F. Weidinger, M. Heger, R. Brooks, C. Stöllberger, C-Y. Ho, L. Perschy, L. Puskas, C. Binder, R. Rosenhek, M. Schneider, M-P. Winter, Belgium:Liege: E. Hoffer, M. Melissopoulou, E. Lecoq, D. Legrand, S. Jacquet, M. Massoz, L. Pierard, S. Marchetta, R. Dulgheru, C. D´ Emal, C. Oury, Jette: S. Droogmans, D. Kerkhove, D. Plein, L. Soens, C. Weytjens, A. Motoc, B. Roosens, I. Lemoine, Edegem: I. Rodrigus, B. Paelinck, B. Amsel, Brussels: P. Unger, D.

Konopnicki, C. Beauloye, A. Pasquet, J.L. Vanoverschelde , S. Pierard, D. Vancraeynest, F. Sinnaeve, Brazil:Sao Paulo: J.L. Andrade, K. Staszko, Porto Alegre: R. Dos Santos Monteiro, M.H. Miglioranza, D.L. Shuha, Rio de Janeiro: M. Alcantara, V. Cravo, L. Fazzio, A. Felix, M. Iso, C. Musa, A.P. Siciliano, Marilia: F. Villaca Filho, A. Rodrigues, F. Vilela, J. Braga, R. Silva, D. Rodrigues, L. Silva, S. Morhy, C. Fischer, R. Silva, M. Vieira, T. Afonso, Fortaleza: J. Abreu, S.N. Falcao, V.A. Moises, A. Gouvea, F.J. Mancuso, A.C. Souza, C.Y. Silva, G. João, C.S. Abboud, R. Bellio de Mattos Barretto, A. Ramos, R. Arnoni, J.E. Assef, D.J. Della Togna, D. Le Bihan, L. Miglioli, A.P. Romero Oliveira, R. Tadeu Magro Kroll, D. Cortez, Belo Horizonte: C.L. Gelape, M.d.C. Peirira Nunes, T.C. De Abreu Ferrari, Canada:Ottawa: K. Hay , Montreal:V. Le, M. Page, F. Poulin, C. Sauve, K. Serri, C. Mercure, Quebec: J. Beaudoin, P. Pibarot, I.A. Sebag, L.G. Rudski, G. Ricafort, Croatia:Zagreb: B. Barsic, V. Krajinovic, M. Vargovic, D. Lovric, V. Reskovic-Luksic, J. Vincelj, S. Jaksic Jurinjak, Cyprus:Nicosia: V. Yiannikourides, M. Ioannides, C. Pofaides, V. Masoura, Czech Republic:Ostrava-Poruba: J. Pudich, Prague: A. Linhart, M. Siranec, J. Marek, K. Blechova, M. Kamenik, Hradec Kralove: R. Pelouch, Zlin: Z. Coufal, M. Mikulica, M. Griva, E. Jancova, M. Mikulcova, Olomouc: M. Taborsky, J. Precek, M. Jecmenova, J. Latal, Liberec: J. Widimsky, T. Butta, S. Machacek, Pilsen: R. Vancata, Brno: J. Spinar, M. Holicka, Ecuador:Guayaquil: F. Pow Chon Long, N. Anzules, A. Bajana Carpio, G. Largacha, E. Penaherrera, D. Moreira, Egypt:Mansoura: E. Mahfouz, E. Elsafty, A. Soliman, Y. Zayed, J. Aboulenein, Alexandria: M. Abdel-Hay, A. Almaghraby, M. Abdelnaby, M. Ahmed, B. Hammad, Y. Saleh, H. Zahran, O. Elgebaly, Zagazig: A. Saad, M. Ali , A. Zeid, R. El Sharkawy, Cairo: A. Al Kholy, R. Doss, D. Osama, H. Rizk, A. Elmogy, M. Mishriky, France:Kremlin-Bicêtre: P. Assayag, S. El Hatimi, Marseille: S. Hubert, J-P. Casalta, F. Gouriet, F. Arregle, S. Cammilleri, L. Tessonnier, A. Riberi, Saint-Etienne: E. Botelho-Nevers, A. Gagneux-Brunon, R. Pierrard, C. Tulane, S. Campisi, J-F. Fuzellier, M. Detoc, T. Mehalla, Nantes: D. Boutoille, A.S. Lecompte, M. Lefebvre, S. Pattier, O. Al Habash, N. Asseray-Madani, C. Biron, J. Brochard, J. Caillon, C. Cueff, T. Le Tourneau, R. Lecomte, M.M. Magali Michel, J. Orain, S. Delarue, M. Le Bras, Limoges: J-F. Faucher, V. Aboyans, A. Beeharry, H. Durox, M. Lacoste, J. Magne, D. Mohty, A. David, V. Pradel, Thonon-les-Bains: V. Sierra, A. Neykova, B. Bettayeb, S. Elkentaoui, B. Tzvetkov, G. Landry, Reims: C. Strady, K. Ainine, S. Baumard, C. Brasselet, C. Tassigny, V. Valente-Pires, M. Lefranc, Pointe-à-Pitre: B. Hoen, B. Lefevre, E. Curlier, C. Callier, N. Fourcade, Brest: Y. Jobic, S. Ansard, R. Le Berre, F. Le Ven, M-C. Pouliquen, G. Prat, P. Le Roux, Rouen: F. Bouchart, A. Savoure, C. Alarcon, C. Chapuzet, I. Gueit, Amiens: C. Tribouilloy, Y. Bohbot, F. Peugnet , M. Gun, Paris: X. Duval, X. Lescure, E. Ilic-Habensus, Nancy: N. Sadoul, C. Selton-Suty, F. Alla, F. Goehringer, O. Huttin, E. Chevalier, Poitiers: R. Garcia, V. Le Marcis, Rennes: P. Tattevin, E. Flecher, M. Revest, Besançon: C. Chirouze, K. Bouiller, L. Hustache-Mathieu, T. Klopfenstein, J. Moreau, D. Fournier, A-S. Brunel, Créteil: P. Lim, L. Oliver, J. Ternacle, A. Moussafeur, Dijon: P. Chavanet, L. Piroth, A. Salmon-Rousseau, M. Buisson, S. Mahy, C. Martins, S. Gohier, Noumea: O. Axler, F. Baumann, S. Lebras, Germany:Bad Oeynhausen: C. Piper, D. Guckel, J. Börgermann, D. Horstkotte, E. Winkelmann, B. Brockmeier, Leipzig: D. Grey, Bonn: G. Nickenig, R. Schueler, C. Öztürk, E. Stöhr, Bad

Nauheim: C. Hamm, T. Walther, R. Brandt, A-C. Frühauf, C.T. Hartung, C. Hellner, C. Wild, Aachen: M. Becker, S. Hamada, W. Kaestner, Berlin: K. Stangl, F. Knebel, G. Baldenhofer, A. Brecht, H. Dreger, C. Isner, F. Pfafflin, M. Stegemann, Ludwigshafen: R. Zahn, B. Fraiture, C. Kilkowski, A-K. Karcher, S. Klinger, H. Tolksdorf, Greece:Athens: D. Tousoulis, C. Aggeli, S. Sideris, E. Venieri, G. Sarri, D. Tsiapras, I. Armenis, A. Koutsiari, G. Floros, C. Grassos, S. Dragasis, L. Rallidis, C. Varlamos, Ioannina: L. Michalis, K. Naka, A. Bechlioulis, A. Kotsia, L. Lakkas, K. Pappas, C. Papadopoulos, S. Kiokas, A. Lioni, S. Misailidou, J. Barbetseas, M. Bonou, C. Kapelios, I. Tomprou, K. Zerva, Voula: A. Manolis, E. Hamodraka, D. Athanasiou, G. Haralambidis, H. Samaras, L. Poulimenos, Hungary:Budapest: A. Nagy, A. Bartykowszki, E. Gara, India:Nagpur: K. Mungulmare, Gurgaon: R. Kasliwal, M. Bansal, S. Ranjan, A. Bhan, Iran:Tehran: M. Kyavar, M. Maleki, F. Noohi Bezanjani, A. Alizadehasl, S. Boudagh, A. Ghavidel, P. Moradnejad, H.R. Pasha, B. Ghadrdoost, Israel:Jerusalem: D. Gilon, J. Strahilevitz, M. Wanounou, S. Israel, Italy:Bari: C. d'Agostino, P. Colonna, L. De Michele, F. Fumarola, M. Stante, Florence: N. Marchionni, V. Scheggi, B. Alterini, S. Del Pace, P. Stefano, C. Sparano, Padova: N. Ruozi, R. Tenaglia, D. Muraru , Grosseto: U. Limbruno, A. Cresti, P. Baratta, M. Solari, Milan: C. Giannattasio, A. Moreo, B. De Chiara, B. Lopez Montero, F. Musca, C.A. Orcese, F. Panzeri, F. Spano, C.F. Russo, O. Alfieri, M. De Bonis, S. Chiappetta, B. Del Forno, M. Ripa, P. Scarpellini, C. Tassan Din, B. Castiglioni , R. Pasciuta, S. Carletti, D. Ferrara, M. Guffanti, G. Iaci, E. Lapenna, T. Nisi, C. Oltolini, E. Busnardo, U. Pajoro, E. Agricola, R. Meneghin, D. Schiavi, Salerno: F. Piscione, R. Citro, R.M. Benvenga, L. Greco, L. Soriente, I. Radano, C. Prota, M. Bellino, D. Di Vece, Genoa: F. Santini, A. Salsano, G.M. Olivieri, Modena: F. Turrini, R. Messora, S. Tondi, A. Olaru, V. Agnoletto, L. Grassi, C. Leonardi, S. Sansoni, Turin: S. Del Ponte, G.M. Actis Dato, A. De Martino, Japan:Nagoya: N. Ohte, S. Kikuchi, K. Wakami, Tsukuba: K. Aonuma, Y. Seo, T. Ishizu, T. Machino-Ohtsuka, M. Yamamoto, N. Iida, H. Nakajima, Tenri: Y. Nakagawa, C. Izumi, M. Amano, M. Miyake, K. Takahashi, Osaka: I. Shiojima, Y. Miyasaka, H. Maeba, Y. Suwa, N. Taniguchi, S. Tsujimoto, Kobe: T. Kitai, M. Ota, Sapporo: S. Yuda, S. Sasaki, Tokyo: N. Hagiwara, K. Yamazaki, K. Ashihara, K. Arai, C. Saitou, S. Saitou, G. Suzuki, Miyazaki: Y. Shibata, N. Watanabe, S. Nishino, K. Ashikaga, N. Kuriyama, K. Mahara, T. Okubo, H. Fujimaki, H. Shitan, H. Yamamoto, K. Abe, M. Terada, S. Takanashi, Tokushima: M. Sata, H. Yamada, K. Kusunose, Y. Saijo, H. Seno, O. Yuichiro, Suita: T. Onishi, F. Sera, S. Nakatani, H. Mizuno, K. Sengoku, Korea, Republic Of:Seoul: S.W. Park, K. Eun Kyoung, L. Ga Yeon, J-w. Hwang, C. Jin-Oh, S-J. Park, L. Sang-Chol, C. Sung-A, S.Y. Jang, R. Heo, S. Lee, J-M. Song, E. Jung, Lithuania:Siauliai: J. Plisiene, A. Dambrauskaite, G. Gruodyte, Kaunas:R. Jonkaitiene, V. Mizariene, J. Atkocaityte, R. Zvirblyte, Luxembourg:Luxembourg: R. Sow, A. Codreanu, T. Staub, C. Michaux, E.C.L. De la Vega, L. Jacobs-Orazi, Malta:Msida: C. Mallia Azzopardi, R.G. Xuereb, T. Piscopo, J. Farrugia, M. Fenech, E. Pllaha, C. Vella, D. Borg, R. Casha, Moldova, Republic Of:Chisinau: L. Grib, E. Raevschi, A. Grejdieru, D. Kravcenco, E. Prisacari, E. Samohvalov, S. Samohvalov, N. Sceglova, E. Panfile, L. Cardaniuc, V. Corcea, A. Feodorovici, V. Gaina, L. Girbu, P. Jimbei, G. Balan, I. Cardaniuc, I. Benesco, V. Marian, N. Sumarga, Montenegro:Podgorica: B. Bozovic, N. Bulatovic, P. Lakovic, L.

Music, Netherlands:Rotterdam: R. Budde, A. Wahadat, T. Gamela, Amsterdam: T. Meijers, Groningen: J.P. Van Melle, V.M. Deursen, Maastricht: H.J. Crijns, S.C. Bekkers, E.C. Cheriex, M. Gilbers, B.L. Kietselaer, C. Knackstedt, R. Lorusso, S. Schalla, S.A. Streukens, Utrecht: S. Chamuleau, M-J. Cramer, A. Teske, T. Van der Spoel, A. Wind, J. Lokhorst, O. Liesbek, H. Van Heusden, The Hague: W. Tanis, I. Van der Bilt, J. Vriend, H. De Lange-van Bruggen, E. Karijodikoro, R. Riezebos, E. van Dongen, J. Schoep, V. Stolk, Norway:Oslo: J.T. Offstad, J.O. Beitnes, T. Helle-Valle, H. Skulstad, R. Skardal, Pakistan:Karachi: N. Qamar, S. Furnaz, B. Ahmed, M.H. Butt, M.F. Khanzada, T. Saghir, A. Wahid, Poland:Warsaw: T. Hryniewiecki, P. Szymanski, K. Marzec, M. Misztal-Ogonowska, Wroclaw: W. Kosmala, M. Przewlocka-Kosmala, A. Rojek, K. Woznicka, J. Zachwyc, Bialystok: A. Lisowska, M. Kaminska, Lodz: J.D. Kasprzak, E. Kowalczyk, D.F. Strzecka, P. Wejner-Mik, Portugal:Carnaxide: M. Trabulo, P. Freitas, S. Ranchordas, G. Rodrigues, Guilhufe: P. Pinto, C. Queiros, J. Azevedo, L. Marques, D. Seabra, Lisbon: L. Branco, J. Abreu, M. Cruz, A. Galrinho, R. Moreira, P. Rio, A.T. Timoteo, M. Selas, V. Carmelo, B. Duque Neves, Almada: H. Pereira, A. Guerra, A. Marques, I. Pintassilgo, Romania:Timisoara: M.C. Tomescu, N-M. Trofenciuc, M. Andor, A. Bordejevic, H.S. Branea, F. Caruntu, L.A. Velcean, A. Mavrea, M.F. Onel, T. Parvanescu, D. Pop, A.L. Pop-Moldovan, M.I. Puticiu, L. Cirin, I.M. Citu, C.A. Cotoraci, D. Darabantiu, R. Farcas, I. Marincu, A. Ionac, D. Cozma, C. Mornos, F. Goanta, I. Popescu, Cluj-Napoca: R. Beyer, R. Mada, R. Rancea, R. Tomoaia, H. Rosianu, C. Stanescu, Russian Federation:Moscow: Z. Kobalava, J. Karaulova, E. Kotova, A. Milto, A. Pisaryuk, N. Povalyaev, M. Sorokina, Saudi Arabia:Jeddah: J. Alrahimi, A. Elshiekh, Riyadh: A. Jamiel, A. Ahmed, N. Attia, Serbia:Belgrade: B. Putnikovic, A. Dimic, B. Ivanovic, S. Matic, D. Trifunovic, J. Petrovic, D. Kosevic, I. Stojanovic, I. Petrovic, P. Dabic, P. Milojevic, Sremska Kamenica: I. Srdanovic, S. Susak, L. Velicki, A. Vulin, M. Kovacevic, A. Redzek, M. Stefanovic, Singapore:Singapore: T.C. Yeo, W. KF Kong, K.K. Poh, Spain:Madrid: I. Vilacosta, C. Ferrera, C. Olmos, M. Abd El- Nasser , Vigo - Pontevedra: F. Calvo Iglesias, E. Blanco-Gonzalez, M. Bravo Amaro, E. Lopez-Rodriguez, J. Lugo Adan, A.N. Germinas, P. Pazos-Lopez, M. Pereira Loureiro, M.T. Perez, S. Raposeiras-Roubin, S. Rasheed Yas, M-M. Suarez-Varela, F. Vasallo Vidal, Barcelona: D. Garcia-Dorado, N. Fernandez-Hidalgo, T. Gonzalez-Alujas, J. Lozano, O. Maisterra, N. Pizzi, R. Rios, Badalona: A. Bayes-Genis, L. Pedro Botet, N. Vallejo, C. Llibre, L. Mateu, R. Nunez, D. Quesada, E. Berastegui, Girona: D. Bosch Portell, J. Aboal Vinas, X. Albert Bertran, R. Brugada Tarradellas, P. Loma-Osorio Ricon, C. Tiron de Llano, Valencia: M.A. Arnau, A. Bel, M. Blanes, A. Osa, Cordoba: M. Anguita, F. Carrasco, J.C. Castillo, J.L. Zamorano, J.L. Moya Mur, M. Alvaro, C. Fernandez-Golfin, J.M. Monteagudo, E. Navas Elorza, Santander: M.C. Farinas Alvarez, J. Aguero Balbin, J. Zarauza, J.F. Gutierrez-Diez, C. Arminanzas , F. Arnaiz de las Revillas, A. Arnaiz Garcia, M. Cobo Belaustegui, M. Fernandez Sampedro, M. Gutierrez Cuadra, L. Garcia Cuello, C. Gonzalez Rico, Barakaldo: R. Rodriguez-Alvarez, J. Goikoetxea , M. Montejo , J.M. Miro, M. Almela, J. Ambrosioni, A. Moreno, E. Quintana, E. Sandoval, A. Tellez, J.M. Tolosana, B. Vidal, C. Falces, D. Fuster, C. Garcia-de-la-Maria, M. Hernandez-Meneses, J. Llopis, F. Marco, I. Ruiz-Zamora, Tarragona: A. Bardaji Ruiz, E. Sanz Girgas, G. Garcia-Pardo, M. Guillen Marzo, A. Rodriguez Oviedo, A. Villares

Jimenez, Tunisia:Sfax: L. Abid, R. Hammami, S. Kammoun, Tunis: M.S. Mourali, F. Mghaieth Zghal, M. Ben Hlima, S. Boudiche, S. Ouali, La Marsa: L. Zakhama, S. Antit, I. Slama, Turkey:Samsun: O. Gulel, M. Sahin, Ankara: L.E. Sade, E. Karacaglar, Istanbul: S. Kucukoglu, O. Cetinarslan, U.Y. Sinan, U. Canpolat, B. Mutlu, H. Atas, R. Dervishova, C. Ileri, United Arab Emirates:Dubai: J. Alhashmi, J. Tahir, P. Zarger, F. Baslib, United Kingdom:London: S. Woldman, L. Menezes, C. Primus, R. Uppal, I. Bvekerwa, Swindon: B. Chandrasekaran, A. Kopanska, J. Chambers, J. Hancock, J. Klein, R. Rajani, M.P. Ursi, S. Cannata, R. Dworakowski, A. Fife, J. Breeze, M. Browne-Morgan, M. Gunning, S. Streather, United States:Washington: F.M. Asch, M. Zemedkun, Uzbekistan:Tashkent: B. Alyavi, J. Uzokov.
